# Heritability and Genome-Wide Association Analyses of Serum Uric Acid in Middle and Old-Aged Chinese Twins

**DOI:** 10.3389/fendo.2018.00075

**Published:** 2018-03-06

**Authors:** Weijing Wang, Dongfeng Zhang, Chunsheng Xu, Yili Wu, Haiping Duan, Shuxia Li, Qihua Tan

**Affiliations:** ^1^Department of Epidemiology and Health Statistics, Public Health College, Qingdao University, Qingdao, China; ^2^Epidemiology and Biostatistics, Department of Public Health, University of Southern Denmark, Odense, Denmark; ^3^Qingdao Municipal Center for Disease Control and Prevention, Qingdao, China; ^4^Qingdao Institute of Preventive Medicine, Qingdao, China; ^5^Unit of Human Genetics, Department of Clinical Research, University of Southern Denmark, Odense, Denmark

**Keywords:** Chinese twins, gene-based test, genome-wide association study, heritability, serum uric acid

## Abstract

Serum uric acid (SUA), as the end product of purine metabolism, has proven emerging roles in human disorders. Here based on a sample of 379 middle and old-aged Chinese twin pairs, we aimed to explore the magnitude of genetic impact on SUA variation by performing sex-limitation twin modeling analyses and further detect specific genetic variants related to SUA by conducting a genome-wide association study. Monozygotic (MZ) twin correlation for SUA level (rMZ = 0.56) was larger than for dizygotic (DZ) twin correlation (rDZ = 0.39). The common effects sex-limitation model provided the best fit with additive genetic parameter (*A*) accounting for 46.3%, common or shared environmental parameter (*C*) accounting for 26.3% and unique/nonshared environmental parameter (*E*) accounting for 27.5% for females and 29.9, 33.1, and 37.0% for males, respectively. Although no SUA-related genetic variants reached genome-wide significance level, 25 SNPs were suggestive of association (*P* < 1 × 10^−5^). Most of the SNPs were located in an intronic region and detected to have regulatory effects on gene transcription. The cell-type specific enhancer of skeletal muscle was detected which has been reported to implicate SUA. Two promising genetic regions on chromosome 17 around rs2253277 and chromosome 14 around rs11621523 were found. Gene-based analysis found 167 genes nominally associated with SUA level (*P* < 0.05), including *PTGR2, ENTPD5*, well-known *SLC2A9*, etc. Enrichment analysis identified one pathway of transmembrane transport of small molecules and 20 GO gene sets involving in ion transport, transmembrane transporter activity, hydrolase activity acting on acid anhydrides, etc. In conclusion, SUA shows moderate heritability in women and low heritability in men in the Chinese population and genetic variations are significantly involved in functional genes and regulatory domains that mediate SUA level. Our findings provide clues to further elucidate molecular physiology of SUA homeostasis and identify new diagnostic biomarkers and therapeutic targets for hyperuricemia and gout.

## Introduction

Serum uric acid (SUA), as the end product of purine metabolism, has proven emerging roles in human disorders, such as kidney disease (1, 2), diabetic nephropathy (3), metabolic diseases (4, 5), preeclampsia (6), cardiovascular disease (2, 7), diabetes (8), etc. A systematic review and meta-analysis by Liu et al. concluded that the prevalence of hyperuricemia (13.3%) and gout (1.1%) was high in mainland China (9). Hence, it is necessary to explore factors affecting SUA homeostasis and elucidate underlying pathogenesis of increased SUA level.

The SUA level is mediated by the interplay between genetic and environmental factors. So far, the magnitude of genetic sources of variance in SUA level has been previously explored in several population studies (10–16). A strong genetic component was indicated with heritability estimates approximately ranging from 35 to 77%. Additionally, the genetic epidemiology has presented an enormous impact on the molecular physiology related to SUA homeostasis by genome-wide association study (GWAS), identifying several genetic loci located in key urate transporters such as *SLC22A7, SLC2A9, SLC22A11, SLC22A12, ABCG2*, etc., and a number of additional intriguing genetic networks (17–19).

Although intensively deployed, no GWAS has, to our knowledge, yet been performed on a sample of middle and old-aged Chinese twins. Chinese population differs in the genetic constitutions and a multitude of life style like dietary habit, work type, and physical activity from other ethnic populations in the world. Genetically related individuals, such as twin pairs, would highly confer increased power in genetic association analysis and efficiently identify genetic variants underlying human complex diseases (20).

Based on a sample of 379 middle and old-aged Chinese twin pairs, we explore the magnitude of genetic impact on SUA variation by performing twin modeling analyses and replicate previous findings on heritability of SUA level and further conduct a GWAS to detect specific genetic variants associated with SUA.

## Materials and Methods

### Participants

The sample collection was carried out through the Qingdao Twin Registry, and details of study recruitment have been described previously (21, 22). Participants who were with gout, systemic lupus erythematosus, eGFR < 60%, or serum creatinine level >1.4 mg/dL were excluded, and incomplete co-twin pairs were also dropped. The final sample consisted of 379 complete twin pairs with a median age of 50 years (95% range: 41–69 years), including 240 monozygotic (MZ) pairs (114 male and 126 female pairs) and 139 dizygotic (DZ) pairs (41 male, 39 female, and 59 opposite-sex pairs).

All co-twin pairs undertook a health examination after a 10–12 h overnight fast and completed a questionnaire. Serum and plasma were separated from blood cells in the field within 30 min and kept frozen at −80°C. The zygosity was determined by using 16 multiple short tandem sequence repeat DNA markers (23, 24). SUA level was measured on the Semi-automatic Analyzer (Hitachi 7600, Japan) and transformed following Blom’s formula for normality.

This study was approved by the Regional Ethics Committee of the Qingdao CDC Institutional Review Boards. Prior written informed consent was achieved for all participants. The ethical principles of the Helsinki Declaration were followed.

### Genotyping and Quality Control

DNA samples of 139 DZ pairs were genotyped on the Illumina’s Infinium Omni2.5Exome-8v1.2 BeadChip platform (Illumina, San Diego, CA, USA). Strong quality control was performed using the genome-wide efficient mixed-model association (GEMMA) (25) by removing the SNPs of call rate (<0.98), Hardy–Weinberg Equilibrium (*P* < 1 × 10^−4^), locus missing (>0.05), and minor allele frequency (<0.05). Finally, a total of 1,365,181 SNPs was included for subsequent GWAS analysis.

### Statistical Analysis

#### Heritability

Data preparation and descriptive analyses as well as genetic analyses were performed with SPSS version 22.0 and Mx program,^1^ respectively. Twin pair phenotypic correlations per zygosity were firstly measured by calculating the Pearson’s product-moment correlation coefficients. The higher correlations of MZ than those of DZ twin pairs indicated the genetic effect on individual differences in SUA level.

Then, standard structural equation modeling methods were used for sex-limitation twin modeling based on the classical twin methods. The variation was decomposed into sources of additive genetic (*A*), common or shared environmental (*C*), and unique/nonshared environmental (*E*) parameters. After the general sex-limitation ACE model was firstly fitted, we then fitted its sub-models: the common effects sex-limitation models by setting the male-specific additive genetic effects (${\text{A}}_{m}^{\prime }$) and sex-specific common or shared environmental effects (*C_m_* and *C_f_*) to 0 and the scalar sex-limitation models by constraining the variance components for females to be equal to a scalar multiple of the variance components for males, respectively.

In order to choose the best fitting model, the likelihood ratio test was applied to compare the performances between the general sex-limitation model and its sub-models. In the likelihood ratio test, twice the difference in the log likelihoods between models was calculated, and change in chi-square against the change in degrees of freedom were tested. The Akaike’s information criterion (AIC) was calculated and a lower AIC indicated a better fit when no statistical difference was observed between two models (26). The covariates of age and body mass index (BMI) were adjusted for in the analysis.

#### Genome-Wide Association Study

##### SNPs-Based Genome-Wide Association Study

The association of SUA level with SNP genotypes was tested using the GEMMA (25). The covariates of age, sex, BMI, and the first five principle components were adjusted for in the model fitting. The conventional genome-wide significance level of *P* < 5 × 10^−8^ and suggestive evidence level of *P* < 1 × 10^−5^ for this association were adopted (27). We further conducted functional elaboration of GWAS results and predicted putative causal variants in haplotype blocks, likely cell types of action and candidate target genes of noncoding genome by using online HaploReg v4.1 software^2^ (28, 29). A set of 25 query SNPs (*P* < 1 × 10^−5^) was submitted. The enrichments of cell-type enhancers with uncorrected *P* < 0.05 were reported.

##### Gene-Based Analysis

We performed gene-based tests on GWAS summary results by using Versatile Gene-based Association Study-2 (VEGAS2) which uses 1,000 genomes data to model SNP correlations across the autosomes and chromosome X (30, 31). In the test, the evidence for association from all SNPs was aggregated within a per gene while correcting for linkage disequilibrium and gene size, and genes showing more signal or strength of association than expected by chance were identified. The SNPs from “1000G East ASIAN Population” were adopted. The *P* < 2.63 × 10^−6^ (0.05/19,001) was considered to be genome-wide significant for the association as 19,001 genes being evaluated.

##### Gene Sets-Based Analysis

A list of significant genes (*P* < 0.05) were included to compute the over-represented gene sets in the gene sets-based analysis using the online version of gene set enrichment analysis (GSEA) program^3^ (32, 33). Gene sets of Canonical pathways, BioCarta, KEGG, Reactome, GO biological process, and GO molecular function were selected in MSigDB. The significance of over-represented gene sets was determined by Benjamini and Hochberg method corrected *P*-value, i.e., false discovery rate (FDR) <0.05.

## Results

### Heritability

The final sample contained a total of 379 twin pairs (240 MZ and 139 DZ pairs) with a median age of 50 years (95% range: 41–69 years). The median (95% range) of SUA level for all participating individuals was 256 µmol/L (143–468 µmol/L), with males having higher SUA level than females [298 (179–509) vs. 226 (130–374), *P* < 0.001] (Table S1 in Supplementary Material).

After adjusting for the effects of age, sex, and BMI, MZ twin correlation for SUA level (*r*_MZ_ = 0.56, 95% CI: 0.47–0.64) was larger than for DZ twin (*r*_DZ_ = 0.39, 95% CI: 0.25–0.50), indicating the presence of genetic influence (Table S2 in Supplementary Material) As described, we firstly fitted the general sex-limitation ACE model and its sub-models and then compared their performances. For the variance in SUA, the common effects sex-limitation model (Model II) provided the best fit (AIC = 443.56, *P* > 0.05) with A parameter accounting for 46.3% (95% CI: 15.0–73.4), *C* parameter accounting for 26.3% (95% CI: 0–55.8), and *E* parameter accounting for 27.5% (95% CI: 19.8–37.9) for females and 29.9% (95% CI: 0–60.0), 33.1% (95% CI: 5.4–63.4), and 37.0% (95% CI: 26.9–50.3) for males, respectively (Table 1).

**Table 1:** Sex-limitation model fitting and proportion of variance for serum uric acid phenotype accounted by genetic and environmental parameters.

Models	A_f_^b^% (95% CI)		*C_f_*% (95% CI)		*E_f_*% (95% CI)		*A_m_*^b^% (95% CI)		*C_m_*% (95% CI)		*E_m_*% (95% CI)		*$A_{m}^{\prime }$%* (95% CI)		−2LL	df	AIC	Compare	ΔLL	Δdf	*P*
Model I (general)	48.74	(15.21–79.03)	23.92	(0–55.68)	27.34	(19.7–37.81)	17.44	(0–59.85)	30.04	(0–63.26)	36.87	(26.77–50.23)	15.64	(0–70.32)	1,937.52	746	445.52	–	–	–	–
Model II^a^ (drop $A_{m}^{\prime }$)	46.29	(15.04–73.36)	26.26	(0–55.84)	27.45	(19.78–37.88)	29.86	(0–59.99)	33.10	(5.43–63.4)	37.04	(26.9–50.32)	–	–	1,937.56	747	443.56	II vs. I	0.05	1	0.832
Model III (drop *C_m_*)	22.28	(0–57.08)	48.57	(16.01–73.33)	29.14	(20.4–47.15)	62.98	(49.8–77.85)	–	–	37.02	(22.15–50.2)	–	–	1,946.97	748	450.97	III vs. II	9.40	1	0.002
Model IV (drop *C_f_*)	72.36	(61.69–80.03)	–	–	27.64	(19.97–38.31)	62.43	(48.42–72.82)	–	–	37.57	(27.18–51.58)	–	–	1,954.68	749	456.68	IV vs. III	7.71	1	0.005
Model V (AE, f = m)	67.91	(60.82–73.77)	–	–	32.09	(26.23–39.18)	67.91	(60.82–73.77)	–	–	32.09	(26.23–39.18)	–	–	1,947.63	751	445.63	V vs. II	10.07	4	0.039
Model VI (ACE, f = m)	67.52	(40.61–73.77)	0.38	(0–25.07)	32.10	(26.23–39.35)	67.52	(40.61–73.77)	0.38	(0–25.07)	32.10	(26.23–39.35)	–	–	1,947.63	750	447.63	VI vs. II	10.07	3	0.018

*Model I: general sex-limitation model*.

*Model II: common effects sex-limitation model, in which the $A_{m}^{\prime }$ was set to 0 based on Model I*.

*Model IIII: common effects sex-limitation model, in which the C_m_ was set to 0 based on Model II*.

*Model IV: common effects sex-limitation model, in which the C_f_ was set to 0 based on Model III*.

*Model V: scalar sex-limitation model, in which females = males for A and E parameters and the C_m_ and C_f_ was set to 0 based on Model II*.

*Model VI: scalar sex-limitation model, in which females = males for all A, C, E parameters based on Model II*.

*^a^The best fitted model, which was chosen on the basis of a change in χ^2^ not representing a significant worsening of fit and a lower AIC indicating a better fit*.

*^b^f, female; m, male*.

*−2LL, −2 Log Likelihood; A, additive genetic influence; $A_{m}^{\prime }$, male-specific additive genetic influence; AIC, Akaike’s information criterion; C, common or shared environmental influence; df, degree of freedom; E, unique/nonshared environmental influence; P, χ^2^ test in model fitting; ΔLL, change in −2 Log Likelihood; Δdf, change in degrees of freedom*.

### Genome-Wide Association Study

**Figure 1:**
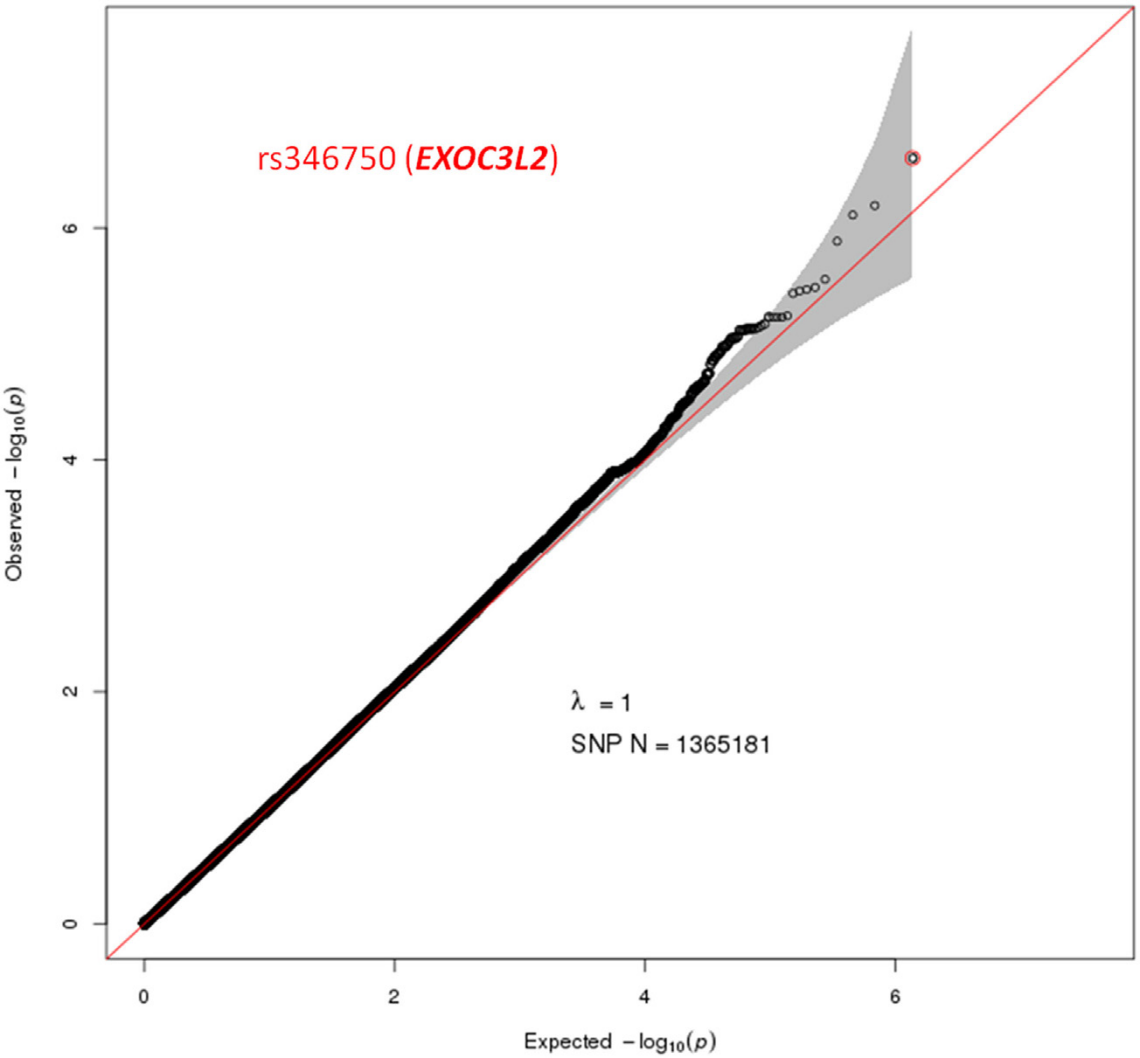
Quantile–quantile plot for quality control check and visualizing crude association for genome-wide association study of serum uric acid (SUA) level. The *x*-axis shows the −log10 of expected *P*-values of association from chi-square distribution and the *y*-axis shows the −log10 of *P*-values from the observed chi-square distribution. The black dots represent the observed data with top hit SNP being colored, and the red line is the expectation under the null hypothesis of no association. Gene at the best SNP is indicated.

**Figure 2:**
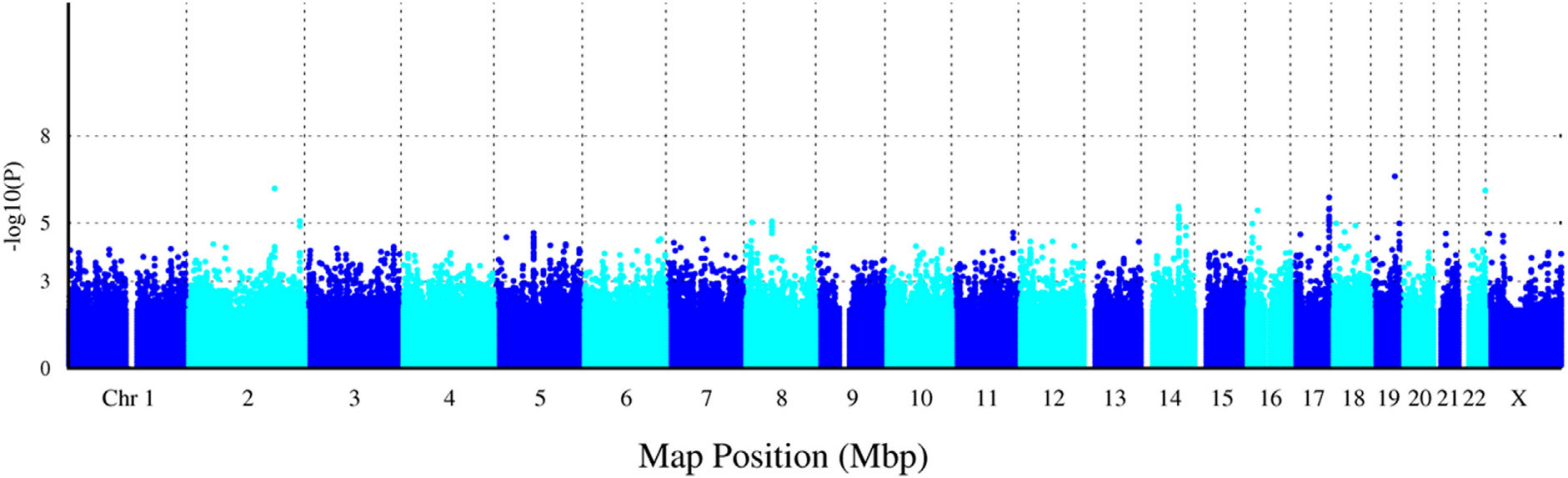
Manhattan plot for genome-wide association study of serum uric acid (SUA) level. The *x*-axis shows the numbers of autosomes and the X chromosome, and the *y*-axis shows the −log10 of *P*-values for statistical significance. The dots represent the SNPs. None of the SNPs reached the genome-wide significance level (*P* < 5 × 10^−8^); however, 25 SNPs were suggestive of association (*P* < 1 × 10^−5^).

A total of 1,365,181 SNPs genotyped from a sample of 139 DZ twin pairs were included for the GWAS of SUA level. The relationship between the observed and expected GWAS *P*-values was illustrated in the Q–Q plot (Figure 1). No evidence of genomic inflation of the test statistics or the bias from the possible population stratification was indicated (λ-statistic = 1). And the slight deviation in the upper right tail from the null distribution suggested evidence for weak association. None of the SNPs reached the genome-wide significance level as illustrated in Figure 2; however, 25 SNPs were suggestive of association (*P* < 1 × 10^−5^) (Table 2). The strongest association was detected with rs346750 (*P* = 2.50 × 10^−7^) in an intronic region of *EXOC3L2* on chromosome 19q13.32.

**Table 2:** The summary of SNPs with *P*-value < 1 × 10^−5^ for association with serum uric acid in genome-wide association study.

SNP	Chr band	CHR	BP	*P*-value	Closest genes or genes	Official full name
rs346750	19q13.32	19	45,737,218	2.50E-07	*EXOC3L2*	Exocyst complex component 3 like 2
rs144505070	22q13.33	22	50,655,722	7.73E-07	*SELENOO*	Tubulin gamma complex associated protein 6
rs2044479	2q31.2	2	179,980,070	6.41E-07	*SESTD1*	SEC14 and spectrin domain containing 1
rs2253277	17q25.3	17	76,109,073	1.30E-06	*TMC6*	Transmembrane channel like 6
					*TNRC6C-AS1*	TNRC6C antisense RNA 1
rs11621523	14q24.3	14	74,307,246	2.77E-06	*PTGR2*	Prostaglandin reductase 2
rs1079120	17q25.3	17	76,092,534	3.25E-06	*TNRC6C*	Trinucleotide repeat containing 6C
kgp8240017 (rs55930513)	14q24.3	14	74,378,876	3.39E-06	*ZNF410*	Zinc finger protein 410
rs61730171	17q25.3	17	76,060,954	3.50E-06	*TNRC6C*	Trinucleotide repeat containing 6C
rs72780857	16p12.3	16	21,096,980	3.68E-06	*DNAH3*	Dynein axonemal heavy chain 3
rs6574154	14q24.3	14	74,396,820	5.73E-06	*ZNF410*	Zinc finger protein 410
rs16970774	17q25.3	17	76,055,547	5.89E-06	*TNRC6C*	Trinucleotide repeat containing 6C
rs16970784	17q25.3	17	76,058,682	5.89E-06	*TNRC6C*	Trinucleotide repeat containing 6C
rs72894061	17q25.3	17	76,048,995	5.89E-06	*TNRC6C*	Trinucleotide repeat containing 6C
rs9893685	17q25.3	17	76,059,784	5.89E-06	*TNRC6C*	Trinucleotide repeat containing 6C
rs4622451	14q24.3	14	74,366,247	6.74E-06	*ZNF410*	Zinc finger protein 410
rs2336742	14q24.3	14	74,436,502	7.02E-06	*ENTPD5*	Ectonucleoside triphosphate diphosphohydrolase 5
rs34293811	17q25.3	17	76,060,866	7.29E-06	*TNRC6C*	Trinucleotide Repeat Containing 6C
rs2159179	14q24.3	14	74,316,848	7.44E-06	*PTGR2*	Prostaglandin reductase 2
rs2270073	14q24.3	14	74,318,754	7.44E-06	*PTGR2*	Prostaglandin reductase 2
rs2302136	14q24.3	14	74,375,956	7.44E-06	*ZNF410*	Zinc finger protein 410
rs2270074	14q24.3	14	74,318,645	7.59E-06	*PTGR2*	Prostaglandin reductase 2
kgp7137390 (rs200828511)	14q24.3	14	74,393,445	7.64E-06	*ZNF410*	Zinc finger protein 410
rs1005564	14q24.3	14	74,410,405	7.64E-06	*FAM161B*	Family with sequence similarity 161 member B
rs2748431	17q25.3	17	76,105,754	8.93E-06	*TNRC6C-AS1*	TNRC6C antisense RNA 1
					*TMC6*	Transmembrane channel like 6
rs1483540	8q11.23	8	54,786,341	8.93E-06	*RGS20*	Regulator of G-protein signaling 20

*kgp, 1000 Genomes Project*.

**Figure 3:**
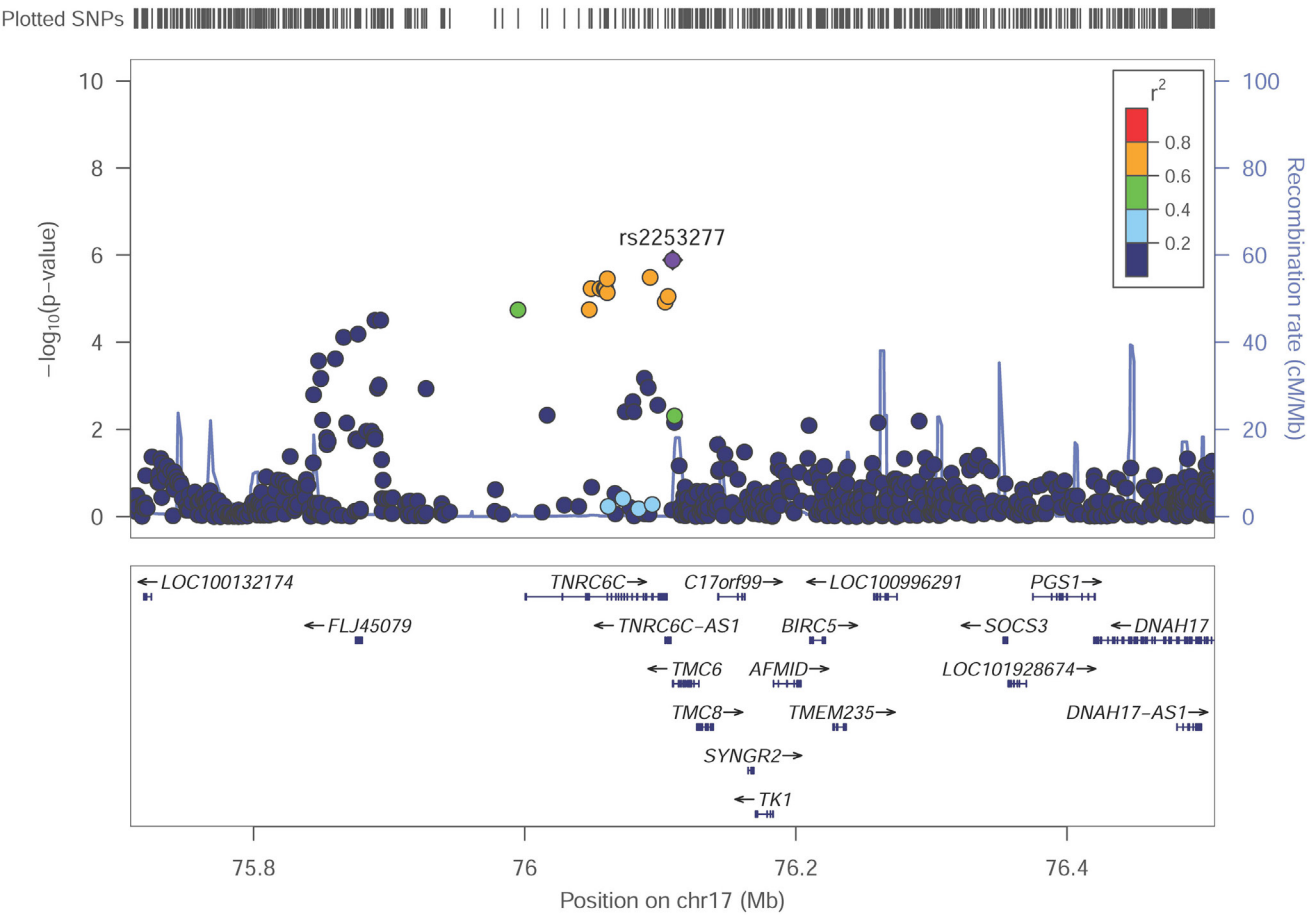
Regional association plot showing signal around chromosomal loci of 17q25.3 for genome-wide association study of serum uric acid (SUA) level. The strongest association was detected with rs2253277 in *TMC6/TNRC6C-AS1* genes.

**Figure 4:**
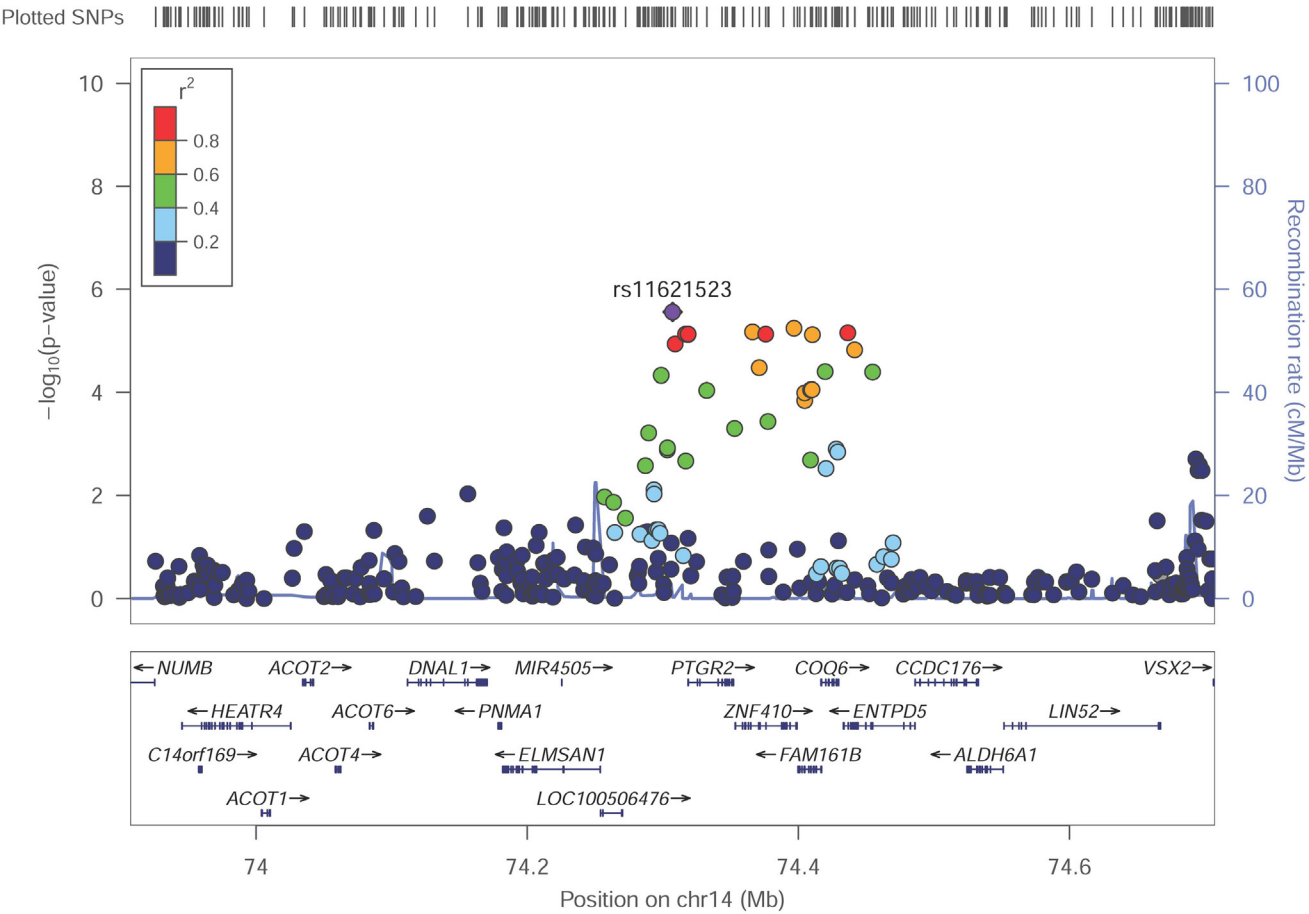
Regional association plot showing signal around chromosomal loci of 14q24.3 for genome-wide association study of serum uric acid (SUA) level. The strongest association was detected with rs11621523 in *PTGR2* gene.

Among these top signals, two chromosomal loci (17q25.3 and 14q24.3) showed nominal association with SUA level as the locus zoom plots illustrated (Figures 3 and 4). On chromosome 17q25.3, seven SNPs (*P* = 3.25 × 10^−6^–7.29 × 10^−6^) and two SNPs (*P* = 1.30 × 10^−6^–8.93 × 10^−6^) were located at or near *TNRC6C* and *TMC6/TNRC6C-AS1* genes, respectively. At chromosome 14q24.3, four SNPs rs2270073, rs2270074, rs11621523, and rs2159179 (*P* = 2.77 × 10^−6^–7.59 × 10^−6^) were positioned within or closest to *PTGR2* gene that was involved in terminal inactivation of prostaglandins. The rs2336742 (*P* = 7.02 × 10^−6^) was located at the intronic region of *ENTPD5* gene, which was involved in the pathway of purine metabolism. The number of SNPs mapping to *ZNF410* and *FAM161B* was five (*P* = 3.39 × 10^−6^–7.64 × 10^−6^) and one (*P* = 7.64 × 10^−6^), respectively. All the abovementioned genes showed nominal association with SUA level (*P* < 0.05) from the following VEGAS2 analysis.

As predicted by HaploReg v4.1, two cell-type specific enhancers (uncorrected *P* < 0.05) of brain angular gyrus (*P* = 0.003) and skeletal muscle (female) (*P* = 0.008) were identified for the set of 25 query SNPs (Table S3 in Supplementary Material). Most of the SNPs were located in intronic regions. Several SNPs were detected within regions with promoter histone marks or enhancer histone marks and could change DNA motifs for DNA-binding proteins, and thus would have regulatory effects on gene transcription (Table S4 in Supplementary Material). We compared previously reported 2,368 significant SUA-associated SNPs in a series of studies with our results. Although no genome-wide significant SNPs were identified in our study, we defined our SNPs with *P* < 0.05 as supportive to the reported SNPs. And 57 SNPs located in genes SLC2A9, ABCG2, LRRC16A, LOC107986260, GLUT9, SCGN, LOC107986971, TFCP2L1, TET2, KCNQ1, FRAS1, SLC16A9, RAF1P1, LOC100129344, LOC107986581, LRRC16A, LOC100287951, WDR1, PDZK1, and LOC107986260 could be replicated (Table S5 in Supplementary Material).

While no genes achieved genome-wide significance level, a total of 167 genes were observed to be nominally associated with SUA level (*P* < 0.05) from VEGAS2 analysis. Genes of *TNRC6C-AS1, ZNF410, TNRC6C, FAM161B, PTGR2, SESTD1, RGS20, ENTPD5, EXOC3L2, DNAH3*, and *TMC6* had already been indicated in the SNPs-based analysis (Table 2), whereas the others were novel. The well-known urate transporter *SLC2A9* gene was also identified. The top 20 genes ranked by *P*-values were listed in Table 3.

**Table 3:** The top 20 genes from Versatile Gene-based Association Study-2 gene-based analysis showing the strongest association with serum uric acid level.

Chr	Gene	Number of SNPs	Start position	Stop position	Gene-based test statistic	*P*-value	Top-SNP	Top-SNP*P*-value
5	*GPR151*	3	145,894,416	145,895,676	32.41	5.00E-06	rs7713676	5.30E-05
17	*TNRC6C-AS1*^a^	2	76,103,482	76,107,880	38.89	1.30E-05	rs2748431	8.90E-06
14	*ZNF410*^a^	9	74,353,317	74,398,991	95.92	1.40E-05	rs6574154	5.70E-06
17	*TNRC6C*^a^	30	76,000,317	76,104,916	253.58	2.30E-05	rs1079120	3.30E-06
5	*JMY*	14	78,531,924	78,623,038	138.00	4.80E-05	rs2591387	3.00E-05
14	*FAM161B*^a^	11	74,399,694	74,417,117	92.90	4.80E-05	rs1005564	7.60E-06
5	*HOMER1*	50	78,669,646	78,809,659	277.24	1.00E-04	rs67994113	3.30E-05
12	*SLCO1B3*	98	20,963,637	21,069,843	712.77	1.20E-04	rs1304539	4.40E-05
14	*PTGR2*^a^	11	74,318,533	74,352,168	59.66	2.20E-04	rs2270073	7.40E-06
10	*C10orf32-ASMT*	18	104,613,966	104,661,655	109.59	2.60E-04	rs10786719	1.70E-04
9	*LAMC3*	88	133,884,503	133,968,446	311.55	3.00E-04	rs10901336	5.40E-04
14	*COQ6*	11	74,416,636	74,429,813	54.92	3.00E-04	rs4903159	4.00E-05
10	*AS3MT*	12	104,629,209	104,661,655	72.58	3.20E-04	rs10786719	1.70E-04
17	*CDRT4*	18	15,339,331	15,370,925	150.75	3.20E-04	rs76787574	2.50E-05
1	*RFX5*	3	151,313,115	151,319,769	25.75	3.30E-04	rs1752387	6.70E-04
10	*NANOS1*	2	120,789,227	120,793,854	21.84	3.30E-04	rs79664216	8.50E-04
17	*EFNB3*	3	7,608,519	7,614,693	33.50	3.40E-04	rs7141	1.40E-04
10	*MYOZ1*	4	75,391,369	75,401,515	32.80	3.40E-04	rs11000726	1.10E-04
10	*EIF3A*	15	120,794,540	120,840,334	98.21	3.40E-04	rs10787901	8.20E-04
19	*EML2*	29	46,112,657	46,148,775	117.77	3.60E-04	rs6509226	1.40E-04

*^a^Represented the genes had already been indicated in the SNPs-based analysis*.

In the gene sets-based analysis using GSEA program, one REACTOME gene set and 20 GO gene sets (FDR *q*-value < 0.01) were presented in Table 4. The only REACTOME pathway was transmembrane transport of small molecules (FDR *q*-value = 0.020). And the GO gene sets were involved in ion transport, regulation of catabolic process, transmembrane transporter activity, hydrolase activity acting on acid anhydrides, cellular process, regulation of immune system process, etc.

**Table 4:** The gene sets results-one REACTOME gene set and top 20 GO gene sets (FDR *q*-value < 0.01) using gene set enrichment analysis (GSEA) program.

Gene set name	Genes in gene set (K)	Description	Genes in overlap (k)	k/K	*P*-value	FDR *q*-value
REACTOME_TRANSMEMBRANE_TRANSPORT_OF_SMALL_MOLECULES	413	Genes involved in transmembrane transport of small molecules	9	0.0218	1.52E-05	2.02E-02
GO_MOVEMENT_OF_CELL_OR_SUBCELLULAR_COMPONENT	1,275	The directed, self-propelled movement of a cell or subcellular component without the involvement of an external agent such as a transporter or a pore	18	0.0141	5.12E-07	1.81E-03
GO_PROTEIN_LOCALIZATION	1,805	Any process in which a protein is transported to, or maintained in, a specific location	21	0.0116	1.25E-06	1.81E-03
GO_REGULATION_OF_INTRACELLULAR_SIGNAL_TRANSDUCTION	1,656	Any process that modulates the frequency, rate or extent of intracellular signal transduction	20	0.0121	1.30E-06	1.81E-03
GO_LOCOMOTION	1,114	Self-propelled movement of a cell or organism from one location to another	16	0.0144	1.84E-06	1.81E-03
GO_REGULATION_OF_MRNA_CATABOLIC_PROCESS	26	Any process that modulates the rate, frequency, or extent of a mRNA catabolic process, the chemical reactions and pathways resulting in the breakdown of RNA, ribonucleic acid, one of the two main type of nucleic acid, consisting of a long, unbranched macromolecule formed from ribonucleotides joined in 3’,5’-phosphodiester linkage	4	0.1538	1.94E-06	1.81E-03
GO_ION_TRANSPORT	1,262	The directed movement of charged atoms or small charged molecules into, out of or within a cell, or between cells, by means of some agent such as a transporter or pore	17	0.0135	2.04E-06	1.81E-03
GO_BIOLOGICAL_ADHESION	1,032	The attachment of a cell or organism to a substrate, another cell, or other organism. Biological adhesion includes intracellular attachment between membrane regions	15	0.0145	3.39E-06	2.59E-03
GO_CATION_TRANSPORT	796	The directed movement of cations, atoms, or small molecules with a net positive charge, into, out of or within a cell, or between cells, by means of some agent such as a transporter or pore	13	0.0163	4.55E-06	3.04E-03
GO_MOTOR_ACTIVITY	131	Catalysis of the generation of force resulting either in movement along a microfilament or microtubule or in torque resulting in membrane scission, coupled to the hydrolysis of a nucleoside triphosphate	6	0.0458	6.83E-06	4.05E-03
GO_NEUROGENESIS	1,402	Generation of cells within the nervous system	17	0.0121	8.14E-06	4.35E-03
GO_POSITIVE_REGULATION_OF_CATABOLIC_PROCESS	395	Any process that activates or increases the frequency, rate, or extent of the chemical reactions and pathways resulting in the breakdown of substances	9	0.0228	1.07E-05	5.19E-03
GO_SMALL_MOLECULE_METABOLIC_PROCESS	1,767	The chemical reactions and pathways involving small molecules, any low molecular weight, monomeric, non-encoded molecule	19	0.0108	1.27E-05	5.53E-03
GO_REGULATION_OF_CELL_ADHESION	629	Any process that modulates the frequency, rate or extent of attachment of a cell to another cell or to the extracellular matrix.	11	0.0175	1.35E-05	5.53E-03
GO_REGULATION_OF_NUCLEAR_TRANSCRIBED_MRNA_CATABOLIC_PROCESS_DEADENYLATION_DEPENDENT_DECAY	15	Any process that modulates the frequency, rate or extent of nuclear-transcribed mRNA catabolic process, deadenylation-dependent decay	3	0.2	1.79E-05	6.14E-03
GO_SECONDARY_ACTIVE_TRANSMEMBRANE_TRANSPORTER_ACTIVITY	233	Catalysis of the transfer of a solute from one side of a membrane to the other, up its concentration gradient. The transporter binds the solute and undergoes a series of conformational changes. Transport works equally well in either direction and is driven by a chemiosmotic source of energy, not direct ATP coupling. Chemiosmotic sources of energy include uniport, symport, or antiport	7	0.03	1.83E-05	6.14E-03
GO_POSITIVE_REGULATION_OF_MRNA_METABOLIC_PROCESS	45	Any process that activates or increases the frequency, rate, or extent of mRNA metabolic process	4	0.0889	1.84E-05	6.14E-03
GO_ENZYME_LINKED_RECEPTOR_PROTEIN_SIGNALING_PATHWAY	689	Any series of molecular signals initiated by the binding of an extracellular ligand to a receptor on the surface of the target cell, where the receptor possesses catalytic activity or is closely associated with an enzyme such as a protein kinase, and ending with regulation of a downstream cellular process, e.g., transcription	11	0.016	3.10E-05	8.97E-03
GO_HYDROLASE_ACTIVITY_ACTING_ON_ACID_ANHYDRIDES	820	Catalysis of the hydrolysis of any acid anhydride	12	0.0146	3.14E-05	8.97E-03
GO_REGULATION_OF_IMMUNE_SYSTEM_PROCESS	1,403	Any process that modulates the frequency, rate, or extent of an immune system process	16	0.0114	3.19E-05	8.97E-03
GO_INTRACELLULAR_SIGNAL_TRANSDUCTION	1,572	The process in which a signal is passed on to downstream components within the cell, which become activated themselves to further propagate the signal and finally trigger a change in the function or state of the cell	17	0.0108	3.48E-05	9.01E-03
GO_ACTIVE_TRANSMEMBRANE_TRANSPORTER_ACTIVITY	356	Catalysis of the transfer of a specific substance or related group of substances from one side of a membrane to the other, up the solute’s concentration gradient. The transporter binds the solute and undergoes a series of conformational changes. Transport works equally well in either direction	8	0.0225	3.68E-05	9.01E-03
GO_CELL_MOTILITY	835	Any process involved in the controlled self-propelled movement of a cell that results in translocation of the cell from one place to another	12	0.0144	3.74E-05	9.01E-03
GO_METAL_ION_TRANSPORT	582	The directed movement of metal ions, any metal ion with an electric charge, into, out of or within a cell, or between cells, by means of some agent such as a transporter or pore	10	0.0172	3.88E-05	9.01E-03

*Collections included CP, CP: BIOCARTA, CP: KEGG, and CP: REACTOME of C2: curated gene sets, and BP and MF of C5: GO gene sets in GSEA*.

*Genes in Overlap (k): the number of genes in the intersection of the query set with a set from MSigDBet*.

*K: the number of genes in the set from MSigDB*.

*FDR q-value: the false discovery rate analog of hypergeometric P-value after correction for multiple hypothesis testing according to Benjamini and Hochberg*.

## Discussion

In this investigation based on 379 twin pairs, we explored the proportion of genetic sources in SUA variation, and confirmed the genetic variants underlying this trait by GWAS. The MZ twin correlation for SUA level was larger than for DZ twin, indicating the presence of genetic influence (Table S2 in Supplementary Material). In fitting the sex-limitation ACE models, the common effects sex-limitation model of dropping ${\text{A}}_{m}^{\prime }$ effects (Model II) was favored over the general model (Model I) (AIC = 443.56, *P* > 0.05), indicating that there was no evidence for sex-specific additive genetic effects. We then considered whether the common or shared environmental effects for males (*C_m_*) (Model III) and further for females (*C_f_*) (Model IV) could also be fixed to 0. However, the goodness-of-fit statistics indicated that these two models provided a significantly worse fit (*P* < 0.05). Thus, these two models were rejected and model II remained the favored one. Finally, we considered the scalar sex-limitation models (Model V and Model VI) by constraining the variance components of females to be equal to scalar multiples of the variance components of males. Both of the models provided a significantly worse fit than Model II (*P* < 0.05). Hence, we concluded that the Model II was the best fitting model, in which additive genetic parameter (*A*) explained larger proportion of SUA variation for females (46.3%) than for males (29.9%), whereas the environmental parameters together (*C* and *E*) explained smaller proportion (53.7 vs. 70.1%) (Table 1).

The sex-difference in the genetic and environmental effects on SUA variation obtained here was in line with the previous Boyle et al.’s genetic study. Based on a sample of 112 twin pairs, they also found a more significant genetic component in control of SUA variation in females than males, whereas a stronger role of environmental component in males (34). We speculated that the genetic architecture may indeed differ across sexes because of the sex differences in selective pressures during human evolution. Additional data from adopted twins and siblings reared together may be used to explore this hypothesis further.

Although no genome-wide significant SNPs were identified in GWAS, we found two promising genetic regions on chromosome 17 around rs2253277 (Figure 3) and chromosome 14 around rs11621523 (Figure 4). The *PTGR2* and *ENTPD5* genes around the rs11621523 have been emphasized for their roles in SUA level. Prostaglandin reductase 2 (PTGR2) is the enzyme involved in terminal inactivation of prostaglandins (35) which may contribute to renal uric acid metabolism (36, 37). Four highly correlated SNPs showing suggestive evidence of association with SUA level were detected within or near *PTGR2* gene. The rs2270073 and rs2270074 were detected within a region with promoter histone marks in the 5’-UTR of *PTGR2* gene and could change DNA motifs for DNA-binding proteins, which provided strong evidence for their regulatory effects on gene transcription (Table S4 in Supplementary Material). Additionally, rs11621523 and rs2159179, which were located in an intergenic region at 14q24.3 and closest to *PTGR2* gene, should also be candidates to be further studied. The protein encoding by *ENTPD5* gene was involved in the pathway of purine metabolism in which SUA was a by-product of oxidation. As SNP rs2336742 was located at the intronic region of *ENTPD5* gene and could change its DNA motifs, it might be associated with purine metabolism as well as SUA level (Table S4 in Supplementary Material). However, the association of novel *TNRC6C, TMC6*, and *TNRC6C-AS1* genes around the other SNP rs225327 with SUA level still needs to be validated. Finally, the enhancer of skeletal muscle was predicted by submitting the set of 25 query SNPs to HaploReg v4.1 (Table S3 in Supplementary Material). And the relationship between SUA level and skeletal muscle strength/volume has been fully researched currently (38–41).

As additional replication, we compared the SUA-associated SNPs reported in a series of studies with ours (18, 42–59) (Table S5 in Supplementary Material). A list of SNPs could be replicated, especially the variants located in the well-known SUA-associated genes SLC2A9 and ABCG2. Notably, two SNPs rs2231142 (ABCG2) and rs10008015 (TET2) were also found by Yang et al. (58) and three SNPs rs11996526 (LOC107986971), rs4848700 (TFCP2L1), and rs179785 (KCNQ1) by Li et al. (49) in Chinese populations.

Even though none genome-wide significant genes were found, a total of 167 genes were observed to be nominally associated with SUA level (*P* < 0.05) from VEGAS2 analysis. The results of GO gene sets from using GSEA program indicated that these genes might be associated with process of generation, catabolism, transport, intracellular signal transduction, and hydrolase activity during SUA metabolism. Besides, several genes were enriched in pathway of transmembrane transport of small molecules, including solute carrier family (*SLC14A2, SLC22A1, SLC2A9, SLC5A3, SLCO1B3*, and *SLC12A5*) and ATPase family (*ATP6V1H* and *ATP11B*), which strengthened their significance in regulating SUA transport process and thus further influencing SUA level (Table 4). Except for the *PTGR2* and *ENTPD5* genes being abovementioned, the *GPR151* gene should be also noted. This gene encodes an orphan member of the class A rhodopsin-like family of G protein-coupled receptors (GPCRs) and thus influences the GPCRs activity. And the GPCRs could regulate the assembly of a multienzyme complex for purine biosynthesis (60). The association of well-known urate transporter gene *SLC2A9* (*P* = 0.001) with SUA level has previously been reported (61, 62). Other genes were of unknown function in terms of SUA level or purine metabolism currently, whereas they may also be interesting potential candidates to be future researched and validated, especially the top 20 genes (Table 3).

Several strengths must be noticed in our study. First, our results based on the twin data of SUA level would be credible. Phenotype variation may be under the effect of subjects’ genetic background, age, gender, and environmental exposures as well as some experimental variables related to sampling, processing, and data analysis. Genetically related individuals, such as twin pairs, would highly confer increased power in genetic association analysis and efficiently identify genetic variants underlying human complex diseases (20). Second, given the various genetic constitutions and multitude of life style among different ethnic populations in the world, this is the first GWAS conducted in the sample of middle and old-aged Chinese twins.

Nevertheless, our study has potential limitations as well. First, our study was with relatively small sample size and limited statistical power resulting from the challenges of identifying and recruiting qualified twin pairs. The results presented here, however, provided a useful reference for hypotheses to be further replicated and validated for exploring increased SUA level. Given the genetic effect on SUA variation is expected to comprise a large number of SNPs possessing very small effect size, a meta-analysis with larger samples will be desirable and ideal. Second, even though we replicated parts of our GWAS results by comparing with results generated from external and independent datasets, most of the SNPs didnot reach the genome-wide significance level. In addition, as no other study has explored the differential expressed genes based on SUA-discordant samples, we cannot yet validate our findings further.

In summary, we have confirmed that genetic factors are significant in explaining SUA level variability through twin modeling. Two novel suggestive regions located at chromosomes 17 and 14 were identified. Twenty-five SNPs reached suggestive evidence level of association with SUA and most of them could have regulatory effects on gene transcription, and 167 genes nominally associated with SUA level were involved in significant biological functions related to uric acid generating and metabolism. The potential candidate biomarkers of SUA level reported here should merit further verifications.

## Data Availability Statements

The SNPs datasets for this study have been deposited in the European Variation Archive (EVA) (Accession No. PRJEB23749).

## Ethics Statement

All participants provided written informed consent for participating in the study which was approved by the local ethics committee at Qingdao CDC, Qingdao, China.

## Author Contributions

DZ and WW contributed to the conception and design. HD and CX organized the collection of samples and phenotypes. WW and YW contributed to sample data and sequencing data management. QT and SL analyzed the sequencing data and WW and CX interpreted the analysis results. WW and DZ drafted the manuscript, YW and HD were involved in the discussion, and QT, SL, and DZ revised it. All the authors read the manuscript and gave the final approval of the version to be published. All the authors agreed to be accountable for all aspects of the work.

## Conflict of Interest Statement

The authors declare that the research was conducted in the absence of any commercial or financial relationships that could be construed as a potential conflict of interest.

## Footnotes

^1^http://www.vcu.edu/mx.

^2^http://archive.broadinstitute.org/mammals/haploreg/haploreg.php.

^3^http://software.broadinstitute.org/gsea/msigdb/annotate.jsp.

## Abbreviations

BMI, body mass index; DZ, dizygotic; GSEA, gene set enrichment analysis; GWAS, genome-wide association study; MZ, monozygotic; SUA, serum uric acid; VEGAS2, Versatile Gene-based Association Study-2.
