# Supplementary material for: Heritability and Genome-Wide Association Analyses of Serum Uric Acid in Middle and Old-Aged Chinese Twins
Source: Front Endocrinol (Lausanne). 2018 Mar 6;9:75. doi: 10.3389/fendo.2018.00075 (PMC5845532; doi:10.3389/fendo.2018.00075)
Supplement: Supplementary file 1 [file Table_1.DOCX]

**Additional file 1: Table S1**. Descriptive statistics for subjects in monozygotic and dizygotic twin pairs

| **Sample** | | **n** | **Age, years** | | |  | **BMI, kg/m^2^** | | |  | **SUA, umol/L** | | |
| --- | --- | --- | --- | --- | --- | --- | --- | --- | --- | --- | --- | --- | --- |
|  |  |  | Range (minimum-maximum) | 2.5%-97.5% Quantiles | Median |  | Range (minimum-maximum) | 2.5%-97.5% Quantiles | Median |  | Range (minimum-maximum) | 2.5%-97.5% Quantiles | Median |
| **MZ** |  |  |  |  |  |  |  |  |  |  |  |  |  |
|  | MZM | 228 | 40 - 80 | 40.00 - 76.00 | 52 |  | 18 - 33 | 18.80 - 30.97 | 23.90 |  | 161 - 651 | 189.40 - 522.20 | 301.00 |
|  | MZF | 252 | 40 - 66 | 41.00 - 64.68 | 50.5 |  | 18 - 38 | 19.03 - 33.87 | 23.50 |  | 93 - 474 | 142.33 - 366.35 | 225.50 |
|  | total | 480 | 40 - 80 | 40.03 - 70.00 | 51 |  | 18 - 38 | 18.99 - 32.41 | 23.70 |  | 93 - 651 | 150.00 - 475.00 | 257.00 |
| **DZ** |  |  |  |  |  |  |  |  |  |  |  |  |  |
|  | DZM | 82 | 41 - 70 | 41.08 - 69.55 | 50 |  | 18 - 33 | 19.33 - 29.38 | 23.90 |  | 143 - 622 | 168.23 - 489.03 | 289.50 |
|  | DZF | 78 | 40 - 70 | 40.00 - 70.00 | 49 |  | 18 - 36 | 19.67 - 32.78 | 23.90 |  | 79 - 514 | 79.98 - 388.23 | 219.00 |
|  | OSDZ | 118 | 41 - 69 | 41.98 - 66.08 | 49 |  | 19 - 33 | 19.59 - 31.22 | 24.75 |  | 120 - 510 | 130.00 - 458.18 | 250.50 |
|  | total | 278 | 40 - 70 | 41.00 - 66.07 | 49 |  | 18 - 36 | 19.70 - 31.20 | 24.10 |  | 79 - 622 | 130.00 - 465.00 | 256.00 |
| **Total** |  |  |  |  |  |  |  |  |  |  |  |  |  |
|  | Male | 369 | 40 - 80 | 41.00 - 72.00 | 50 |  | 18 - 33 | 19.01 - 30.58 | 24.10 |  | 143 - 651 | 179.23 - 508.88 | 298.00 |
|  | Female | 389 | 40 - 70 | 41.00 - 65.00 | 50 |  | 18 - 38 | 19.02 - 32.78 | 23.70 |  | 79 - 514 | 130.00 - 373.50 | 226.00 |
|  | total | 758 | 40 - 80 | 41.00 - 69.03 | 50 |  | 18 - 38 | 19.10 - 31.63 | 23.90 |  | 79 - 651 | 143.00 - 468.30 | 256.00 |

**Note**: BMI, body mass index; DZ, dizygotic pairs; DZF, female dizygotic pairs; DZM, male dizygotic pairs; MZ, monozygotic pairs; MZF, female monozygotic pairs; MZM, male monozygotic pairs; OSDZ, opposite sex dizygotic pairs; SUA, serum uric acid
