# Supplementary material for: Heritability and Genome-Wide Association Analyses of Serum Uric Acid in Middle and Old-Aged Chinese Twins
Source: Front Endocrinol (Lausanne). 2018 Mar 6;9:75. doi: 10.3389/fendo.2018.00075 (PMC5845532; doi:10.3389/fendo.2018.00075)
Supplement: Supplementary file 2 [file Table_2.DOCX]

**Additional file 2: Table S2**. Phenotypic correlation coefficients (95% confidence intervals) with covariates' effects in MZ and DZ twin pairs

| **Model** | **MZ** | |  | **DZ** | | **-2LL** | **df** | **χ^2^** | ***P*-value** |
| --- | --- | --- | --- | --- | --- | --- | --- | --- | --- |
|  | corr. | (95%CI) |  | corr. | (95%CI) |  |  |  |  |
| Base | 0.56 | (0.47-0.64) |  | 0.39 | (0.25-0.50) | 1772.02 | 749 | - | - |
| Drop sex | 0.68 | (0.61-0.74) |  | 0.34 | (0.20-0.46) | 1947.63 | 750 | 175.61 | < 0.001* |
| Drop age | 0.56 | (0.47-0.64) |  | 0.39 | (0.25-0.50) | 1772.26 | 750 | 0.24 | 0.628 |
| Drop BMI | 0.58 | (0.48-0.65) |  | 0.36 | (0.23-0.48) | 1818.90 | 750 | 46.88 | < 0.001* |

**Note**: χ^2^, difference of χ^2^ value; -2LL, -2 Log Likelihood; BMI, body mass index; corr., correlation coefficient; df, degree of freedom; DZ, dizygotic; MZ, monozygotic
