# Supplementary material for: Heritability and Genome-Wide Association Analyses of Serum Uric Acid in Middle and Old-Aged Chinese Twins
Source: Front Endocrinol (Lausanne). 2018 Mar 6;9:75. doi: 10.3389/fendo.2018.00075 (PMC5845532; doi:10.3389/fendo.2018.00075)
Supplement: Supplementary file 4 [file Table_4.DOCX]

**Additional file 4: Table S4**. The results for a set of 25 query SNPs (*P* < 1×10^-5^) in HaploReg v4.1

| **SNP** | **chr** | **pos (hg38)** | **Ref** | **Alt** | **ASN freq** | **Promoter histone marks** | **Enhancer histone marks** | **Proteins bound** | **Motifs changed** | **Selected eQTL hits** | **GENCODE genes** | **dbSNP func annot** |
| --- | --- | --- | --- | --- | --- | --- | --- | --- | --- | --- | --- | --- |
| rs346750 | 19 | 45233960 | A | C | 0.19 | ESC, IPSC, SKIN | ESC, SKIN, ADRL | - | STAT | 2 hits | *EXOC3L2* | intronic |
| rs144505070 | 22 | 50217293 | C | T | 0 | - | LIV | POL2 | - | - | *SELENOO* | missense |
| rs2044479 | 2 | 179115343 | G | A | 0.3 | - | - | - | 5 altered motifs | 10 hits | *SESTD1* | intronic |
| rs2253277 | 17 | 78112992 | C | T | 0.1 | GI | 17 tissues | 4 bound proteins | - | 7 hits | *TMC6* | 3'-UTR |
| rs11621523 | 14 | 73840543 | C | A | 0.53 | SKIN | - | - | - | 50 hits | *PTGR2* | - |
| rs1079120 | 17 | 78096453 | T | A,C,G | 0.14 | - | - | - | - | 4 hits | *TNRC6C* | intronic |
| kgp8240017 (rs55930513) | 14 | 73912173 | T | C | - | - | ADRL | - | 7 altered motifs | - | *ZNF410* | intronic |
| rs61730171 | 17 | 78064873 | A | C | 0.14 | - | BRST, BRN, MUS | - | 5 altered motifs | 5 hits | *TNRC6C* | synonymous |
| rs72780857 | 16 | 21085659 | A | G | 0.08 | - | GI, LIV | - | Ik-2 | - | *DNAH3* | intronic |
| rs6574154 | 14 | 73930117 | C | A | 0.64 | - | - | - | Foxp1, Mef2 | 33 hits | *ZNF410* | intronic |
| rs16970774 | 17 | 78059466 | G | A | 0.13 | MUS | 13 tissues | - | AhR, HNF4, Hmx | 5 hits | *TNRC6C* | intronic |
| rs16970784 | 17 | 78062601 | A | T | 0.14 | - | HRT, MUS, BLD | GATA1 | HP1-site-factor, Mrg, Tgif1 | 1 hit | *TNRC6C* | intronic |
| rs72894061 | 17 | 78052914 | G | A | 0.13 | - | - | - | RORalpha1, RXRA | 3 hits | *TNRC6C* | intronic |
| rs9893685 | 17 | 78063703 | A | G | 0.13 | - | FAT, BLD | - | Myc, SREBP | 4 hits | *TNRC6C* | intronic |
| rs4622451 | 14 | 73899544 | A | G | 0.63 | - | 4 tissues | - | RP58, Spdef | 32 hits | *ZNF410* | intronic |
| rs2336742 | 14 | 73969799 | G | A | 0.56 | - | SKIN | - | CHOP::CEBPalpha | 51 hits | *ENTPD5* | intronic |
| rs34293811 | 17 | 78064785 | C | G | 0.13 | - | BRST, BRN, MUS | - | EWSR1-FLI1 | 5 hits | *TNRC6C* | missense |
| rs2159179 | 14 | 73850145 | C | T | 0.57 | - | 4 tissues | - | Nkx3 | 51 hits | *PTGR2* | - |
| rs2270073 | 14 | 73852051 | G | T | 0.57 | 24 tissues | - | 19 bound proteins | LUN-1, Osr, ZNF263 | 49 hits | *PTGR2* | 5'-UTR |
| rs2302136 | 14 | 73909253 | A | C | 0.57 | - | - | - | Cdx2, Pdx1, Pou5f1 | 52 hits | *ZNF410* | intronic |
| rs2270074 | 14 | 73851942 | G | C | 0.58 | 24 tissues | - | 31 bound proteins | FXR, LXR | 50 hits | *PTGR2* | 5'-UTR |
| kgp7137390 (rs200828511) | 14 | 73926742 | G | T | - | - | - | - | 6 altered motifs | - | *ZNF410* | intronic |
| rs1005564 | 14 | 73943702 | T | C | 0.64 | - | - | - | GATA, TCF4 | 32 hits | *FAM161B* | intronic |
| rs2748431 | 17 | 78109673 | G | A | 0.13 | - | 5 tissues | - | AP-1, AP-2 | 6 hits | *TNRC6C-AS1* | - |
| rs1483540 | 8 | 53873781 | G | A | 0.3 | - | SKIN | - | Irf, Pax-5, SIX5 | - | *RGS20* | intronic |
