# Supplementary material for: Heritability and Genome-Wide Association Analyses of Serum Uric Acid in Middle and Old-Aged Chinese Twins
Source: Front Endocrinol (Lausanne). 2018 Mar 6;9:75. doi: 10.3389/fendo.2018.00075 (PMC5845532; doi:10.3389/fendo.2018.00075)
Supplement: Supplementary file 5 [file Table_5.DOCX]

**Additional file 5: Table S5**. The comparison between our results of genome-wide association study and other previously reported serum uric acid-associated SNPs

| **SNP** | **Chr** | **Our study*** | |  | **Other studies** | | | | | |
| --- | --- | --- | --- | --- | --- | --- | --- | --- | --- | --- |
|  |  | Beta | *P*-value |  | Reference | Population | Sample size | Trait/disease | Beta | *P*-value |
| ***SLC2A9*** |  |  |  |  |  |  |  |  |  |  |
| rs16890979 | 4 | -0.644 | 2.20E-02 |  | Kottgen, et al. | European ancestry | >140,000 | Serum urate | -0.378 | 0 |
|  |  |  |  |  | Chen, et al. | American | 8,998 | Serum uric acid | -0.352 | 2.64E-59 |
|  |  |  |  |  | Yang, et al. | American | > 7000 | Serum uric acid | NA | 1.60E-76 |
|  |  |  |  |  | Cummings, et al. | Non-European | 388 | Serum urate | NA | 1.31E-02 |
|  |  |  |  |  | Chen, et al. | American | 9,000 | Serum uric acid | Negatively | 1.20E-20 |
|  |  |  |  |  | McArdle, et al. | American | 868 | Serum uric acid | 0.430 | 7.89E-09 |
|  |  |  |  |  | Dehghan, et al. | American | 7,699 | Serum uric acid | -0.360 | 1.60E-76 |
| rs1014290 | 4 | -0.276 | 1.35E-03 |  | Kottgen, et al. | European ancestry | >140,000 | Serum urate | 0.355 | 0 |
|  |  |  |  |  | Nakayama, et al. | Japanese | 2158 | Gout | 1.800^#^ | 1.58E-15 |
|  |  |  |  |  | Li, et al. | European-American | 1,060 | Plasma uric acid | NA | 1.13E-09 |
|  |  |  |  |  | Charles, et al. | African American | 1,017 | Serum uric acid | -0.150 | 8.68E-07 |
|  |  |  |  |  | Vitart, et al. | Croatian | 794 | Serum uric acid | NA | 2.80E-05 |
| rs13129697 | 4 | -0.245 | 3.05E-03 |  | Kottgen, et al. | European ancestry | >140,000 | Serum urate | 0.354 | 0 |
|  |  |  |  |  | Voruganti, et al. | Hispanic children | 815 | Serum uric acid | NA | NA |
|  |  |  |  |  | Li, et al. | European-American | 1,060 | Plasma uric acid | NA | 1.12E-10 |
|  |  |  |  |  | Karns, et al. | European descent | 1,300 | Serum uric acid | -28.990^#^ | 2.33E-19 |
| rs3775948 | 4 | -0.306 | 4.14E-04 |  | Kottgen, et al. | European ancestry | >140,000 | Serum urate | 0.356 | 0 |
|  |  |  |  |  | Matsuo, et al. | Japanese | 2,158 | Gout | 1.640^#^ | 6.70E-15 |
|  |  |  |  |  | Charles, et al. | African Americans | 1,017 | Serum uric acid | -0.183 | 1.38E-09 |
| rs737267 | 4 | -0.644 | 2.20E-02 |  | Kottgen, et al. | European ancestry | >140,000 | Serum urate | -0.365 | 0 |
|  |  |  |  |  | Li, et al. | European-American | 1,060 | Plasma uric acid | NA | 7.34E-06 |
|  |  |  |  |  | Vitart, et al. | Croatian | 770 | Serum uric acid | NA | 8.60E-06 |
| rs7442295 | 4 | -0.644 | 2.20E-02 |  | Kottgen, et al. | European ancestry | >140,000 | Serum urate | 0.385 | 0 |
|  |  |  |  |  | Cummings, et al. | Non-European | 380 | Serum urate | NA | 1.24E-02 |
|  |  |  |  |  | Wallace, et al. | White European | 1,955 | Serum urate | -0.020 | 1.85E-15 |
| rs7675964 | 4 | -0.256 | 2.05E-03 |  | Kottgen, et al. | European ancestry | >140,000 | Serum urate | -0.352 | 0 |
|  |  |  |  |  | Voruganti, et al. | Hispanic children | 815 | Serum uric acid | NA | NA |
| rs6855911 | 4 | -0.644 | 2.20E-02 |  | Kottgen, et al. | European ancestry | >140,000 | Serum urate | 0.364 | 0 |
|  |  |  |  |  | Cummings, et al. | Non-European | 388 | Serum urate | NA | 6.30E-03 |
| rs10939650 | 4 | -0.258 | 2.75E-03 |  | Kottgen, et al. | European ancestry | >140,000 | Serum urate | 0.356 | 0 |
|  |  |  |  |  | Charles, et al. | African Americans | 1,017 | Serum uric acid | -0.153 | 4.23E-07 |
| rs4447863 | 4 | 0.205 | 1.66E-02 |  | Kottgen, et al. | European ancestry | >140,000 | Serum urate | 0.210 | 0 |
|  |  |  |  |  | Li, et al. | European-American | 1,060 | Plasma uric acid | NA | 5.06E-06 |
| rs938552 | 4 | -0.644 | 2.20E-02 |  | Voruganti, et al. | Hispanic children | 815 | Serum uric acid | NA | NA |
|  |  |  |  |  | Cummings, et al. | Non-European | 391 | Serum urate | NA | 2.64E-02 |
| rs3733590 | 4 | -0.300 | 7.84E-04 |  | Kottgen, et al. | European ancestry | >140,000 | Serum urate | 0.105 | 5.30E-17 |
| rs6849736 | 4 | -0.295 | 9.81E-04 |  | Kottgen, et al. | European ancestry | >140,000 | Serum urate | -0.106 | 3.10E-17 |
| rs3733589 | 4 | -0.295 | 9.81E-04 |  | Nakayama, et al. | Japanese | 2,158 | Gout | 1.480^#^ | 2.00E-07 |
| rs10023068 | 4 | -0.251 | 3.97E-03 |  | Kottgen, et al. | European ancestry | >140,000 | Serum urate | -0.313 | 0 |
| rs6853437 | 4 | -0.251 | 3.97E-03 |  | Kottgen, et al. | European ancestry | >140,000 | Serum urate | 0.313 | 0 |
| rs13144899 | 4 | -0.242 | 6.44E-03 |  | Kottgen, et al. | European ancestry | >140,000 | Serum urate | -0.122 | 5.10E-19 |
| rs10805346 | 4 | 0.203 | 1.83E-02 |  | Kottgen, et al. | European ancestry | >140,000 | Serum urate | 0.267 | 0 |
| rs10939665 | 4 | 0.199 | 3.49E-02 |  | Kottgen, et al. | European ancestry | >140,000 | Serum urate | -0.185 | 6.40E-238 |
| rs1122141 | 4 | 0.203 | 1.97E-02 |  | Kottgen, et al. | European ancestry | >140,000 | Serum urate | 0.199 | 1.40E-245 |
| rs11722229 | 4 | -0.644 | 2.20E-02 |  | Kottgen, et al. | European ancestry | >140,000 | Serum urate | 0.382 | 0 |
| rs13125646 | 4 | -0.644 | 2.20E-02 |  | Kottgen, et al. | European ancestry | >140,000 | Serum urate | -0.365 | 0 |
| rs10006397 | 4 | -0.222 | 1.05E-02 |  | Kottgen, et al. | European ancestry | >140,000 | Serum urate | 0.307 | 0 |
| rs6449144 | 4 | 0.237 | 3.89E-02 |  | Kottgen, et al. | European ancestry | >140,000 | Serum urate | 0.209 | 8.10E-271 |
| rs734553 | 4 | -0.644 | 2.20E-02 |  | Kottgen, et al. | European ancestry | >140,000 | Serum urate | 0.383 | 0 |
| rs7654258 | 4 | -0.187 | 3.16E-02 |  | Kottgen, et al. | European ancestry | >140,000 | Serum urate | -0.099 | 1.10E-19 |
| rs7678012 | 4 | 0.185 | 3.81E-02 |  | Kottgen, et al. | European ancestry | >140,000 | Serum urate | -0.190 | 2.40E-250 |
| rs998675 | 4 | 0.200 | 2.21E-02 |  | Kottgen, et al. | European ancestry | >140,000 | Serum urate | -0.209 | 3.30E-288 |
| rs9998811 | 4 | -0.644 | 2.20E-02 |  | Kottgen, et al. | European ancestry | >140,000 | Serum urate | -0.390 | 0 |
| ***ABCG2*** |  |  |  |  |  |  |  |  |  |  |
| rs2231142 | 4 | 0.277 | 2.94E-03 |  | Kottgen, et al. | European ancestry | >140,000 | Serum urate | 0.221 | 4.40E-116 |
|  |  |  |  |  | Yang, et al. | Chinese | 3,451 | Serum uric aicd | 0.046 | 1.19E-14 |
|  |  |  |  |  | Karns, et al. | European descent | 1,300 | Serum uric aicd | 27.4^#^ | 5.14E-06 |
|  |  |  |  |  | Chen, et al. | American | 8,998 | Serum uric aicd | 0.246 | 1.46E-15 |
|  |  |  |  |  | Yang, et al. | American | > 7,000 | Serum uric aicd | NA | 9.00E-20 |
|  |  |  |  |  | Chen, et al. | American | 9,000 | Serum uric aicd | Positively | 8.30E-09 |
|  |  |  |  |  | Dehghan, et al. | American | 7,699 | Serum uric aicd | 0.25 | 9.00E-20 |
|  |  |  |  |  | Huffman, et al. | European descent | 42,741 | Serum urate | -0.223 (Lean individuals); -0.215 (Overweight individuals) | 1.55E-29 (Lean individuals); 1.55E-30 (Overweight individuals) |
| rs1871744 | 4 | -0.202 | 3.16E-02 |  | Nakayama, et al. | Japanese | 2,158 | Gout | 1.93^#^ | 3.85E-14 |
| rs4693924 | 4 | 0.193 | 4.55E-02 |  | Kottgen, et al. | European ancestry | >140,000 | Serum urate | 0.171 | 8.50E-63 |
| rs4148157 | 4 | 0.193 | 4.55E-02 |  | Kottgen, et al. | European ancestry | >140,000 | Serum urate | 0.17 | 7.60E-62 |
| rs2054576 | 4 | 0.193 | 4.55E-02 |  | Kottgen, et al. | European ancestry | >140,000 | Serum urate | -0.175 | 3.70E-64 |
| ***LRRC16A*** |  |  |  |  |  |  |  |  |  |  |
| rs12183240 | 6 | -0.214 | 2.81E-02 |  | Kottgen, et al. | European ancestry | >140,000 | Serum urate | -0.035 | 2.10E-08 |
| rs17253044 | 6 | -0.467 | 2.43E-02 |  | Kottgen, et al. | European ancestry | >140,000 | Serum urate | -0.05 | 6.90E-11 |
| rs1980450 | 6 | -0.243 | 1.24E-02 |  | Kottgen, et al. | European ancestry | >140,000 | Serum urate | 0.034 | 5.00E-08 |
| rs3788994 | 6 | -0.558 | 5.80E-03 |  | Kottgen, et al. | European ancestry | >140,000 | Serum urate | -0.047 | 6.30E-11 |
| rs742132 | 6 | -0.224 | 2.28E-02 |  | Kottgen, et al. | European ancestry | >140,000 | Serum urate | 0.035 | 1.90E-08 |
| ***LOC107986260*** |  |  |  |  |  |  |  |  |  |  |
| rs7656072 | 4 | -0.218 | 1.07E-02 |  | Kottgen, et al. | European ancestry | >140,000 | Serum urate | 0.069 | 9.30E-31 |
| rs7697246 | 4 | -0.178 | 4.05E-02 |  | Kottgen, et al. | European ancestry | >140,000 | Serum urate | -0.147 | 2.80E-131 |
| ***LOC100287951*** |  |  |  |  |  |  |  |  |  |  |
| rs4610325 | 4 | -0.185 | 3.17E-02 |  | Kottgen, et al. | European ancestry | >140,000 | Serum urate | 0.148 | 5.50E-131 |
| rs7661365 | 4 | -0.186 | 3.13E-02 |  | Kottgen, et al. | European ancestry | >140,000 | Serum urate | 0.067 | 5.70E-09 |
| ***Other genes*** |  |  |  |  |  |  |  |  |  |  |
| rs16892419  (*GLUT9*) | 4 | -0.213 | 1.49E-02 |  | Kottgen, et al. | European ancestry | >140,000 | Serum urate | -0.082 | 4.10E-08 |
|  |  |  |  |  | Shin, et al. | Korean | 8,834 | Serum urate | NA | 9.00E-03 |
| rs2294344  (*SCGN*) | 6 | -0.669 | 1.48E-04 |  | Kottgen, et al. | European ancestry | >140,000 | Serum urate | -0.046 | 1.50E-09 |
| rs11996526  (*LOC107986971*) | 8 | 0.518 | 3.90E-03 |  | Li, et al. | Chinese | 3,103 | Gout arthritis | 1.85^#^ | 4.96E-05 |
| rs4848700  (*TFCP2L1*) | 2 | 0.229 | 6.89E-03 |  | Li, et al. | Chinese | 3,103 | Gout arthritis | 0.63^#^ | 7.93E-07 |
| rs10008015  (*TET2*) | 4 | 0.29 | 3.47E-02 |  | Yang, et al. | Chinese | 3,451 | Serum uric acid | 0.012 | 9.98E-06 |
| rs179785  (*KCNQ1*) | 11 | -0.204 | 1.97E-02 |  | Li, et al. | Chinese | 3,103 | Gout arthritis | 0.66^#^ | 2.86E-05 |
| rs6845871  (*FRAS1*) | 4 | 0.201 | 1.43E-02 |  | Li, et al. | European-American | 1,060 | Serum uric acid | NA | 3.88E-05 |
| rs1171658  (*SLC16A9*) | 10 | -0.211 | 2.35E-02 |  | Kottgen, et al. | European ancestry | >140,000 | Serum urate | -0.041 | 3.40E-10 |
| rs11732042  (*RAF1P1*) | 4 | 0.333 | 3.84E-02 |  | Kottgen, et al. | European ancestry | >140,000 | Serum urate | 0.078 | 4.60E-30 |
| rs11947517  (*LOC100129344*) | 4 | -0.176 | 4.41E-02 |  | Kottgen, et al. | European ancestry | >140,000 | Serum urate | 0.157 | 3.90E-144 |
| rs12216125  (*LOC107986581*) | 6 | 0.253 | 3.96E-02 |  | Kottgen, et al. | European ancestry | >140,000 | Serum urate | 0.041 | 1.30E-11 |
| rs1980450  (*LRRC16A*) | 6 | -0.243 | 1.24E-02 |  | Kottgen, et al. | European ancestry | >140,000 | Serum urate | 0.034 | 5.00E-08 |
| rs6834555  (*WDR1*) | 4 | -0.186 | 3.85E-02 |  | Kottgen, et al. | European ancestry | >140,000 | Serum urate | 0.239 | 1.90E-264 |
| rs9728619  (*PDZK1*) | 1 | 0.175 | 3.47E-02 |  | Kottgen, et al. | European ancestry | >140,000 | Serum urate | 0.058 | 3.10E-21 |
| rs9990427  (*LOC107986260*) | 4 | -0.18 | 3.64E-02 |  | Kottgen, et al. | European ancestry | >140,000 | Serum urate | 0.066 | 2.30E-09 |

**Note:** * We performed the genome-wide association study on serum uric acid in a sample of 139 middle and old-aged Chinese dizygotic twin pairs

^#^ The beta value was not available, but the odds ratio or effect size value was provided

Chr, chromosome; NA, not available
